# Tissue factor pathway inhibitor-2 (TFPI-2)—an underappreciated partaker in cancer and metastasis

**DOI:** 10.1007/s10555-024-10205-7

**Published:** 2024-08-17

**Authors:** Marek Z. Wojtukiewicz, Marta Mysliwiec, Anna Tokajuk, Joanna Kruszewska, Barbara Politynska, Anmbreen Jamroze, Anna M. Wojtukiewicz, Dean G. Tang, Kenneth V. Honn

**Affiliations:** 1 Department of Oncology, Medical University of Bialystok, 12 Ogrodowa, 15-027 Bialystok, Poland; 2 Department of Clinical Oncology, Comprehensive Cancer Center of Bialystok, 12 Ogrodowa, 15-027 Bialystok, Poland; 3 Department of Psychology and Philosophy, Medical University of Bialystok, 37 Szpitalna, 15-295 Bialystok, Poland; 4 Robinson College, University of Cambridge, Grange Road, Cambridge, CB3 9AN UK; 5 Department of Pharmacology and Therapeutics, Roswell Park Comprehensive Cancer Center, Buffalo, NY 14263 USA; 6 Department of Pathology-School of Medicine, Bioactive Lipids Research Program, Wayne State University, 540 East Canfield Avenue, Detroit, MI 48201 USA; 7 Karmanos Cancer Institute, 4100 John R St, Detroit, MI 48201 USA; 8 Department of Chemistry, Wayne State University, 5101 Cass Ave, Detroit, MI 48202 USA

**Keywords:** Tissue factor pathway inhibitor, Coagulation, Hemostasis, Cancer, Metastasis, Tumor

## Abstract

The coagulation system is known to play an important role in cancer development and metastasis, but the precise mechanisms by which it does so remain incompletely understood. With this in mind, we provide an updated overview of the effects of TFPI-2, a protease inhibitor, on cancer development and metastasis. TFPI-2 interacts with the thrombin cascade and also employs other mechanisms to suppress cancer growth and dissemination, which include extracellular matrix stabilization, promotion of caspase-mediated cell apoptosis, inhibition of angiogenesis and transduction of intracellular signals. Down-regulation of TFPI-2 expression is well documented in numerous types of neoplasms, mainly via promoter methylation. However, the exact role of TFPI-2 in cancer progression and possible approaches to up-regulate TFPI-2 expression warrant further studies. Strategies to reactivate TFPI-2 may represent a promising direction for future anticancer studies and therapy development.

## Introduction

Tissue factor pathway inhibitor-2 (TFPI-2) is a structural homolog of tissue factor pathway inhibitor (TFPI), an endogenous inhibitor of tissue-factor-dependent blood coagulation [1]. It is a multivalent Kunitz-type serine protease inhibitor that has biological functions distinct from TFPI. This review focuses on the role of TFPI-2 in cancer biology.

## Structure and functions of TFPI-2

TFPI-2 belongs to the superfamily of serine protease inhibitors that contain one or more Kunitz-type domains [2]. It was originally isolated from placental tissue [3, 4] and rediscovered in 1994 when its homologous cDNA was isolated [5, 6]. The human *TFPI-2* gene maps to chromosome 7 in the 7q22 region, which consists of five exons and four introns [7]. Mature TFPI-2 protein has a short acidic N-terminal region, three tandem Kunitz inhibitor domains, and a C-terminal basic region, and depending on the nature of glycosylation, presents in three forms: 27 kDa, 31 kDa and 33 kDa [6, 8]. The N-terminal Kunitz domain 1 of TFPI-2 is endowed with inhibitory activity on serine proteases while the two domains (first and second domain) of TFPI-1, downstream of the Kunitz domain, exert inhibitory effects on FVIIa/TF and FXa, respectively [9].

Despite its structural homology, the biological functions of TFPI-2 differ from those of TFPI-1, namely the latter is the principal inhibitor of tissue factor – dependent blood coagulation, whereas TFPI-2 serves as a weak inhibitor of factor VIIa-tissue factor (VIIa-TF) complex. The interaction of TFPI-2 with VIIa-TF is enhanced by heparin [10]. The second Kunitz – type domain of the TFPI-2 molecule does not bind factor Xa, unlike TFPI-1 [10]. It has been shown that TFPI-2 expresses a weak inhibitory activity towards cathepsin G and factor IXa-poly(lysine) (indirectly inhibiting fXa in a muted manner). Incubation of TFPI-2 (in the absence of heparin) with urokinase and tissue-type plasminogen activator, along with thrombin, does not produce any significant inhibition of the proteins [10]. However, recombinant TFPI-2 exhibits a strong inhibitory effect towards the amidolytic activities of serine proteinases including plasmin, plasma kallikrein (pKLK), trypsin and chymotrypsin, as well as factor XIa [10]. TFPI-2 inhibits proteases via the P1 arginine residue (Arg-24) in its first Kunitz – type domain [11, 12]. TFPI-2 fails to inhibit glandular kallikrein, urinary plasminogen activator, tissue plasminogen activator, human activated protein C, leukocyte elastase and thrombin [10]. Although TFPI-2 is present in blood at a low level, its impact on blood coagulation in healthy individuals appears to be minimal. Despite this, in pregnancy the serum concentration of TFPI-2 increases up until the end of gestation, when it reaches > 10 nM, thus potentially becoming a significant player in the inhibition of coagulation in certain physiological and pathological conditions [13, 14].

Interestingly, the TFPI-2 C-terminal peptide—EDC34 has been shown to possess a broad range of antimicrobial activity against Gram-negative bacterial pathogens by binding with IgG, IgA, IgE, and IgM immunoglobulins. The interaction between EDC34 and the Fc part of IgG enhances the effector functions of factor C1q, leading to the activation of a classical complementary pathway during infection, of Gram-negative bacterial etiology [15]. A specific host defense mechanism of TFPI-2 against invading Gram-negative bacterial pathogens has been reported with the consequent therapeutic implications [15].

A growing body of evidence indicates that TFPI-2 is a relevant tumor suppressor [16] which is related to its regulation of extracellular matrix (ECM) and fundamental to normal development as well as tumor invasion/metastasis [17–19], atherosclerosis [20] and chronic inflammation [21, 22]. TFPI-2 has been shown to function as a mitogen, which augments mitogen-activated protein kinase (MAPK) and subsequently c-fos expression, thus promoting the signal transduction pathway of MAPK/extracellular signal-regulated kinase (ERK) from surface receptors to nuclear processes, leading to mitosis of vascular smooth muscle cells. TFPI-2 is also an established growth factor for retinal pigment epithelial cells [23, 24]. However, TFPI-2 has been reported to act in an antiangiogenic fashion by exerting a direct inhibitory effect on ECs [25]. In fact, adenoviral-mediated TFPI-2 gene transfer inhibited the growth of a human glioblastoma (GBM) cell line [26] and laryngeal squamous cell carcinoma in a nude mouse model [27]. Others have also reported that TFPI-2 induces apoptosis and inhibits angiogenesis [28]. TFPI-2 is synthesized by ECs in the vasculature and the majority of the protein is secreted into the extracellular matrix [29]. TFPI-2 inhibits a wide variety of serine proteinases including plasmin, trypsin and elastase. Plasmin, through direct and indirect activation of matrix metalloproteinases (MMPs), promotes ECM degradation [30], which is important for tumor invasion and metastasis. Markedly reduced or undetectable expression of TFPI-2 has been demonstrated in tumors originating from various tissue types. On the one hand, this may be related to gene silencing via promoter hypermethylation, while on the other, aberrantly spliced TFPI-2 transcript has also been observed in tumor cells [31].

## TFPI-2 expression and levels in normal and neoplastic tissue

### TFPI-2 localization under normal conditions

TFPI-2 is mainly synthesized in ECs of small blood vessels [29] and less so in other blood vessels (venous, arterial), keratinocytes, dermal fibroblasts, placenta, liver, heart, kidney, smooth muscle cells [5], and synovioblasts [32] TFPI-2 is expressed in most human tissues, such as skeletal muscles, pancreas, placenta, colon, stomach, brain, esophagus and ovaries [5, 33–35]. TFPI-2 protein has been detected in cytotrophoblasts and syncytiotrophoblasts, as well as in seminal vesicles, breast, larynx, endometrium and brain tissue [36, 37]. Most (up to 90%) synthesized TFPI-2 is secreted into the ECM [29]. Despite the fact that the TFPI-2 level in blood is barely detectable (0.43–0.49 ng/mL), it can be found in preovulatory follicular fluid, the mucous of the uterus and seminal plasma [38–41].

Tests performed on normal human and atherosclerotic arteries have shown that in healthy tissues TFPI-2 was detected only in the vascular endothelium, whereas in atherosclerotic arteries it was also found in macrophages, T cells and smooth muscles, as well as vascular ECs. The TFPI-2 antigen was found in both plasma membranes and in the ECM. Interestingly, TFPI-2 was detected in atheroma, in close proximity to tissue factor and factor VII, and also to plasmin/plasminogen on macrophages and on ECs, suggesting that TFPI-2 may be significant to the functional regulation of plasmin activity and proteolytic mechanisms in atherosclerotic lesions [42].

Although the TFPI-2 plasma levels in healthy subjects are low and may be incapable of significantly affecting coagulation, platelets contain high concentrations of TFPI-2 (fourfold higher than in plasma), which can be secreted during platelet activation to inhibit fibrinolysis induced by tissue-type plasminogen activator. It has been suggested that TFPI-2 derives from megakaryocytes [13]. Platelet α-granules may contain TFPI-2 and release it, which *in vitro* appears to contribute to platelet-mediated inhibition of clot lysis [13].

### TFPI-2 expression and levels in neoplastic tissues

Research over the last 20 years has demonstrated that TFPI-2 protein levels are very low or undetectable in many tumor cells compared to normal cells. TFPI-2 is downregulated in many aggressive tumors, such as gliomas [43], non-small cell lung cancer (NSCLC) [44, 45], breast cancer (BC) [46], melanoma [47], colorectal cancer (CRC) [48], pancreatic cancer (PC) [49], hepatocellular carcinoma (HCC) [50], gastric stromal tumor, cervical cancer [51], and prostate cancer [52]. Interestingly, the expression of TFPI-2 decreases with increasing degree of malignancy in multiple neoplasms (gliomas, breast, colon, pancreatic, laryngeal, endometrial and renal cancers) [33, 53].

In human cancers, expression of TFPI-2 appears to be upregulated only in clear cell carcinoma (CCC) tissues [54]. The diagnostic usefulness of the determination of TFPI-2 in the serum of patients with epithelial ovarian cancer (EOC), especially CCC has been established [14].

As far as the central nervous system is concerned, high levels of TFPI-2 have been confirmed in normal brain tissue, whereas gliomas and anaplastic astrocytomas express greatly reduced amounts. In GBM, TFPI-2 has consistently been untraceable [53]. This finding has been replicated in NSCLCs in which TFPI-2 gene expression was decreased 4- to 120-fold compared to normal lung tissues [30, 44]. Likewise, the expression of TFPI-2 is low in most HCC [50]. By contrast, high levels of TFPI-2 expression have been detected in lobular carcinoma of the breast, in one of the BC types [33]. Similarly, in urothelial cancer, the TFPI-2 level was found to be inversely correlated with increasing grade and stage of cancer [55]. The same study indicated that TFPI-2 may have a pro-apoptotic role and be negatively related to the proliferative index (Ki-67), which is an indicator of the aggressiveness of bladder and other cancers [55]. Low TFPI-2 expression levels have been linked to cancer progression, recurrence and poor survival. The negative associations between TFPI-2 levels and tumor malignancy and progression have been reported for decades. For more information, please refer to our earlier publication on the role of TFPI-2 in malignancy [33, 56].

**Fig. 1:**
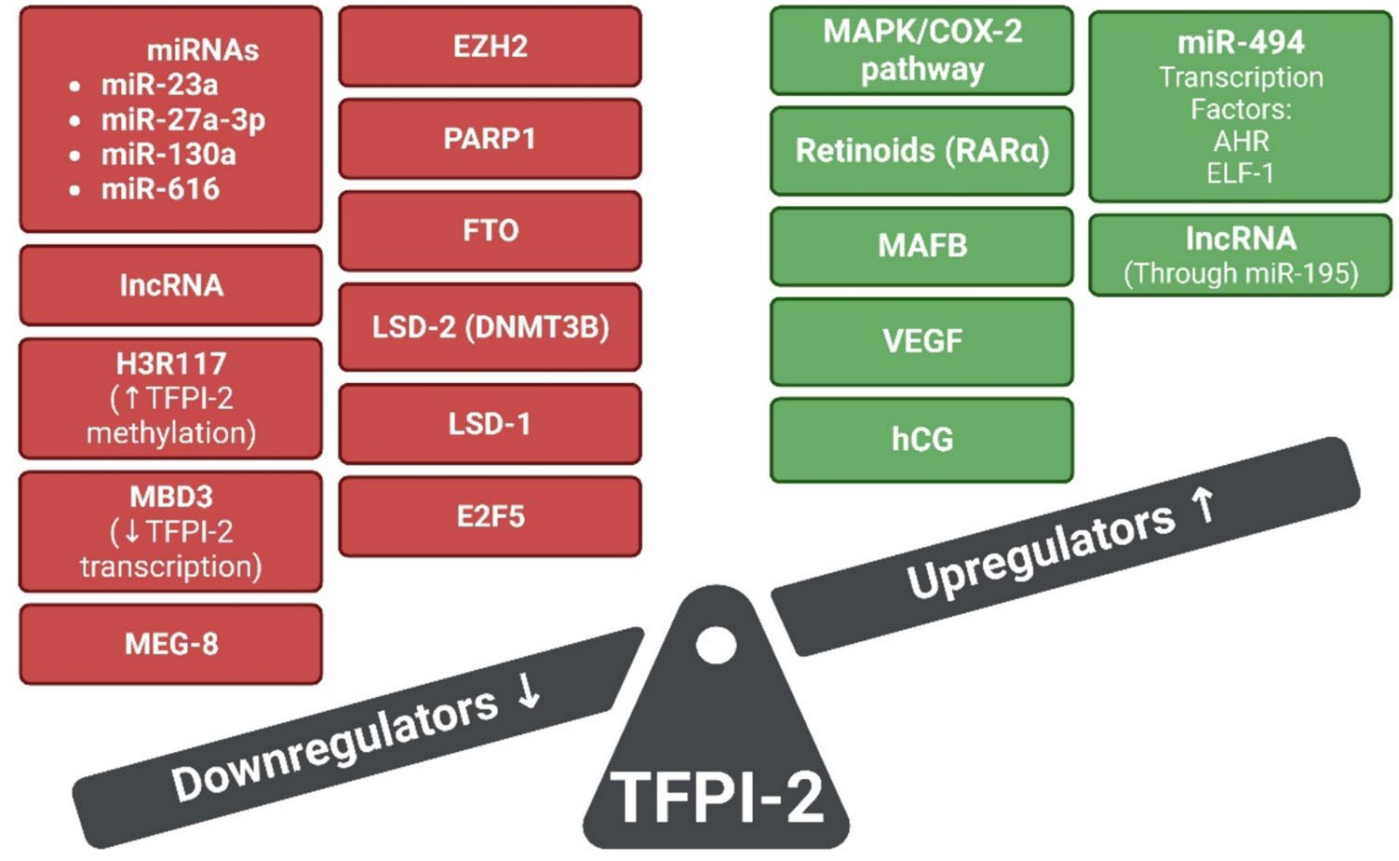
Regulators of TFPI-2 expression and function. Red boxes represent compounds that downregulate TFPI-2 expression or function, while green boxes represent compounds that upregulate TFPI-2 expression or function. Created with BioRender.com. (COX-2—cyclooxygenase-2; EZH2—zeste homolog 2; FTO—fat mass and obesity-related protein; lncRNAs—long non-coding RNAs; LSD1—lysine specific demethylase 1; LSD2—lysine specific demethylase 2; MAFB—musculo-aponeurotic fibrosarcoma oncogene homolog B; MAPK—mitogen-activated protein kinase; MBD3—methyl-CpG binding domain protein 3; MEG8—maternally expressed gene 8; miRNAs—microRNA; PARP1—Poly (ADP-ribose) polymerase; RARα—retinoid acid receptor α; VEG—vascular endothelial growth factor)

Regulatory mechanisms of TFPI-2 expression are summarized in Fig. 1.

## The role of TFPI-2 in malignancy—beyond coagulation

### Interactions with hemostasis impacting cancer development

Generally, activation of the coagulation cascade is related to cancer dissemination and progression to the systemic stage [57–61]. Tissue factor (TF) is the major factor in clotting activation and forms the axis of the relation between clotting and tumor development [57, 62]. It has been demonstrated that higher TF levels are indicative of marked invasiveness and metastasis [57, 63](Rao 1992, Zhang, Deng et al. 1994). TFPIs play a pivotal role in TF-VIIa inactivation [6, 64]. TFPI limits the TF-dependent coagulation cascade, and the formation of factor Xa and thrombin. TFPI-2 acts as another, but weak inhibitor of the TF-dependent pathway. Nevertheless, the precise significance of its effects remains elusive.

Down-regulation of the TF pathway has been proposed as one of the mechanisms of TFPI-2 regulatory functions. The TF activity enhances thrombin generation, which subsequently leads to increased TFPI-2 production [65], for example in human liver myofibroblasts [66]. The upregulation of TFPI-2 was found to require the catalytic activity of thrombin, which was confirmed by eliminating it with hirudin. Furthermore, protease-activated receptor-1 (PAR-1) blocking antibody was found to reverse the effect of thrombin. Interestingly, thrombin promoted the expression of cyclooxygenase-2 (COX-2) mRNA by the MAPK-related pathway which was abolished by a specific COX-2 inhibitor. In conclusion, thrombin enhances TFPI-2 synthesis via the MAPK/COX-2 pathway through PAR1-signaling. Thrombin-mediated up-regulation of TFPI-2 may be a self-limiting mechanism, since TFPI-2 in turn limits thrombin generation and blood coagulation. Additionally, when thrombin promotes ECM degradation via inducing matrix metalloproteinases, TFPI-2 also takes part in this regulation indirectly by means of limiting thrombin activity [65].

Thrombin thus seems to be a double-edged sword. On the one hand, it takes part in the coagulation cascade, while on the other, it may limit the extrinsic pathway of blood coagulation and support ECM maintenance, thus having an impact on suppressing cancer development [56, 67] (described below). Since thrombin formation leads to activation of platelets, TFPI-2 indirectly down-regulates platelet aggregation and activation and thus lowers its ability to support cancer development [56].

Further, TFPI-2 limits the activity of PARs (particularly PAR-1 and PAR-4) by limiting thrombin formation and formation of the fXa/TF/fVIIa complex. There is well established evidence that pronounced activation of PARs induces tumor growth, angiogenesis, and improves tumor cells’ adhesive potential for platelets, ECs and fibronectin. The pleiotropic role of PARs in cancer has been thoroughly reviewed previously by Wojtukiewicz et al. [67, 68].

**Fig. 2:**
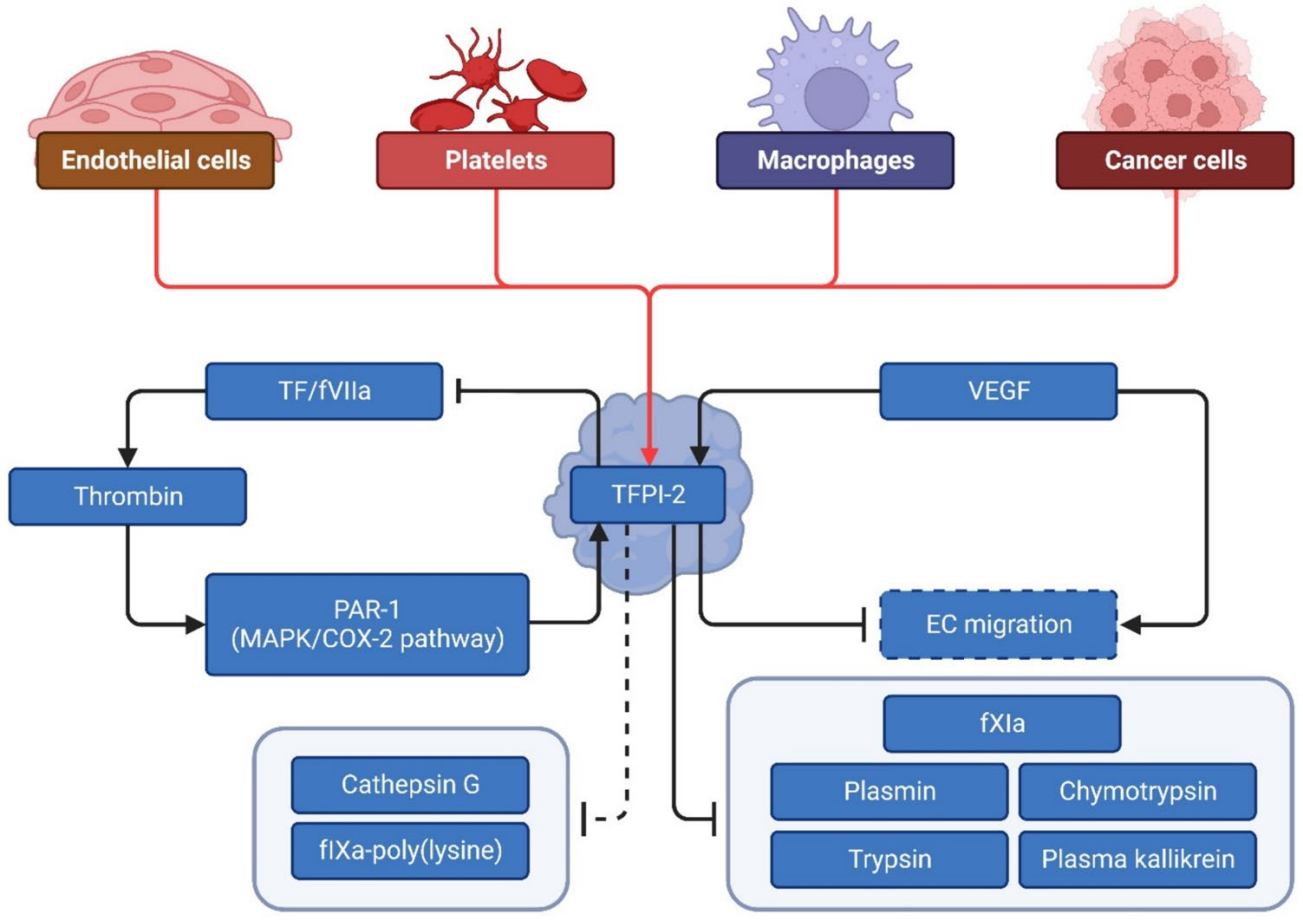
Interaction of TFPI-2 with blood coagulation. Versatile sources of TFPI-2 protein include endothelial cells, platelets, macrophages, and cancer cells. Created with BioRender.com. (COX-2—cyclooxygenase-2; ECs—endothelial cells; fVIIa—factor VIIa; fXIa—factor XIa; MAPK—mitogen-activated protein kinase; PAR-1—protease-activated receptor-1; TF—tissue factor; TFPI-2—Tissue factor pathway inhibitor-2; VEGF—vascular endothelial growth factor

Interactions of TFPI-2 with selected elements of the coagulation system and factors related to cancer development are depicted in Fig. 2.

### Interaction of TFPI-2 with cancer—mechanisms beyond hemostasis

#### Endothelial proliferation

TFPI-2 expression in ECs is up-regulated by vascular endothelial growth factor (VEGF) and represents a form of negative feedback regulation [25]. VEGF caused a time- and dose-dependent elevation in TFPI-2 both at the mRNA and protein levels in ECs. Simultaneously, IL-1β significantly increased TFPI-2 mRNA expression. Up-regulation of TFPI-2 was also found in the context of tumor necrosis factor (TNF)-α and fibroblast growth factor (FGF)-2, but only slightly [25]. VEGF and FGF-2 stimulate EC proliferation (via a mechanism dependent on AKT and ERK1/2 pathways), which was blocked by TFPI-2 protein, but not the first Kunitz-type domain of TFPI-2 (KD1). Since KD1 is responsible for the proteinase inhibitory activity of TFPI-2, this suggests that the antiproliferative effect of TFPI-2 is more complex than just its proteinase-inhibitory effects. Additionally, at higher doses, TFPI-2 inhibited VEGF receptor-2 (VEGFR2) activity [25]. This is dependent on MEK1/2 kinase associated with ERK1/2 activation, which is consistent with ERK-dependent *TFPI-2* gene activation in other cell types [65, 69]. Considering that VEGF-dependent induction of EC proliferation is related to ERK signaling, ERK suppression may underlie TFPI-2 mediated inhibition of EC proliferation.

Furthermore, inflammatory mediators, such as lipopolysaccharide and TNF-α, mildly upregulate *TFPI-2* gene expression in endothelium [29]. Similarly, IL-1β mediates upregulation of *TFPI-2* in endothelium during the acute phase of inflammation (> tenfold increase of TFPI-2) [25].

#### Tumor microenvironment (TME)

TFPI-2 stabilizes the TME, thus limiting the development of cancer. Over the last 20 years, considerable research has attempted to clarify the role of TFPI-2 in coagulation and malignancy. Research in cell lines has revealed that TFPI-2 is correlated with lower invasiveness of GBM SNB19 [43, 70] human lung cancer A549 [71], prostate cancer LNCaP [52], fibrosarcoma HT-1080 [72], pancreatic ductal adenocarcinoma [49], gastric cancer (GC) [73], BC [74]), amelanotic melanoma C-32 [75] and thyroid cancer [76] cells. In experimental models of highly invasive human GBM, TFPI-2 was undetectable, in contrast to normal expression in healthy brain and lower-grade brain tumors [33, 43].

TFPI-2 may also induce apoptosis [77, 78], as described in TFPI-2-positive GBM cell lines (SNB-19) and low-grade gliomas (Hs683) [77]. Interestingly, restoration of TFPI-2 expression in human GBM (U-251) promoted both intrinsic and extrinsic caspase-dependent (caspase-9 and caspase-3) activation of pro-apoptotic signals, and inhibited angiogenesis and invasion [78]. 

A recent study by Lavergne et al. identified TFPI-2 as a potent inhibitor of KLK5 and KLK12 [79]. Computer modeling suggests that the first Kunitz domain of TFPI-2 may mediate interactions with KLK12. As KLK12 could activate proMMP-1 and -3, inhibition of KLK12 by TFPI-2 might limit MMP activation and inhibit cancer progression [79].

Tumor invasion and metastasis are accompanied by ECM degradation and gradual replacement of original tissue stroma by tumor-derived ECM [80]. As an inhibitor of MMPs, TFPI-2 restricts matrix degradation promoted by invading tumor cells. In 2011, Gaud et al. showed (using NCI-H460 NSCLC cell line and a nude mice orthotopic model) that stable *TFPI-2* down-regulation caused an increase of α1 integrin on the cell surface, thus enhancing cell adhesion to collagen IV and laminin, increasing MMP-1 and MMP-3 expression, and promoting cancer cell invasion through the basement membrane. Additionally, they showed that incubation of pulmonary fibroblasts with conditioned media from cancer cells with silenced *TFPI-2* increased MMP expression and promoted lung cancer cell invasion and metastases [81]. TFPI-2 functions as a potent inhibitor of plasmin and down-regulates plasmin-mediated proteolysis of ECM; consequently, up-regulation of TFPI-2 counteracts the ECM degradation by cancer cells [82, 83]. It has been reported that TFPI-2 diminishes plasmin- and trypsin-mediated activation of pro-MMP-1 and pro-MMP-3, leading to decreased ECM degradation and invasion of HT-1080 fibrosarcoma cells [72].

Similarly, TFPI-2 expression level is negatively related to the progression of bladder cancer. TFPI-2 expression in muscle-invasive bladder cancer was lower than in non-muscle-invasive bladder cancer (*P* < 0.05), and its expression was also lower in lymph node metastatic disease. In TFPI-2 overexpressing cells, the invasion of bladder cancer BIU-87 cells was significantly reduced *in vitro*. Further experiments indicated that MMP-1 expression was higher in bladder cancer cells than normal adjacent tissue and in particular, higher in muscle-invasive than in non-muscle-invasive bladder cancer and that MMP-1 mRNA and protein levels were inhibited significantly in BIU-87-TFPI-2 cells [84].

TFPI-2 inhibits multiple MMPs including MMP-1, MMP-2, MMP-9 and MMP-13, thus playing a pivotal role in ECM regulation [20]. Both TFPI-2 overexpression and exogenous rTFPI-2 (recombinant TFPI-2) inhibited proliferation and invasion of breast cancer cells. Co-culture of MCF7 cells with a medium of increasing concentrations of human rTFPI-2 dose-dependently inhibited MCF7 cell proliferation. It is worth noticing that TFPI-2 was detected either in cytoplasm or in the nucleus and its over-expression was associated with greater nuclear translocation. TFPI-2 overexpression led to decreased nuclear expression of MMP-2 mRNA and protein, an effect mediated through the formation of TFPI-2/AP-2α nuclear complex and down-regulation of MMP-2 transcription [85].

#### Angiogenesis

TFPI-2 has been reported to be an angiogenesis inhibitor [86]. In addition to the negative effects on EC-mediated angiogenesis, TFPI-2 also increases caspase-mediated cell apoptosis [87]. The ECs of small blood vessels secrete TFPI-2 and release it into the ECM [29], which helps maintain ECM integrity and basement membrane stability, thus preventing the initiation of angiogenesis. Notably, VEGF up-regulates TFPI-2 synthesis in endothelium whereas TFPI-2 suppresses EC proliferation through a negative-feedback mechanism [25]. It is of interest that in experimental melanoma, TFPI-1 and -2 inhibit vasculogenic mimicry (VM) [88], during which aggressive tumor cells form vessel-like networks without typical ECs to supply sufficient blood to the growing tumor [89]. TFPI-2 is required for MMP-2 activity and essential for VM, while TFPI-1 plays a role in the appropriate perfusion of blood vessels newly formed through VM [88]. On the other hand, TFPI-2 was found to be up-regulated when melanoma-associated antigen 3 (MAGEA3) was silenced leading to impaired VM, with the implication that the influence of TFPI-2 on VM in different cancers requires further investigation [90].

Cancer cells disseminate through the process called angiotropism, in which they attach to and flow along blood vessels and metastasize. In the melanoma *in vivo* model, high levels of TFPI-2 have been correlated with worse outcomes in uveal melanoma (UM), but with better outcomes in cutaneous melanoma (CM) [91]. This ‘paradox’ might be related to observations that in UM, the TFPI-2 co-expressed genes are correlated with pathways of human papilloma virus (HPV) infection and calcium signaling whereas in CM, the TFPI-2 co-expressed genes are correlated with cytokine–cytokine receptor interaction and TNF signaling [91]. Many new studies have continued to define the mechanisms of TFPI-2 mediated inhibition of angiogenesis, and some have concluded that the anti-angiogenic effects of TFPI-2 are not based on the inhibition of proliferation [92]. Instead, TFPI-2, localized in the ECM, appears to restrain angiogenesis via inhibiting MMP-1, -2, -9 and -13 and plasmin and preventing matrix degradation. Some other studies also suggest that the Maternally Expressed Gene 8 (MEG8)/TFPI-2 axis may contribute to ECM remodeling and regulation of angiogenesis [92, 93].

#### TFPI-2 as a tumor suppressor gene. Genetic and epigenetic regulation of TFPI-2 expression and function

The *TFPI-2* gene is mapped on chromosome 7q21.3. In support of its functioning as a tumor suppressor, *TFPI-2* is epigenetically silenced in aggressive cancers including, among others, colorectal cancer or CRC [48, 94], glioma [43], NSCLC [44], pancreatic cancer or PC [49], BC [46], melanoma [47], HCC [50], and oral squamous cell carcinoma (OSCC) [95]. Hypermethylation of the *TFPI-2* gene promoter was higher in metastatic cancer in contrast to localized tumors and under-expression of TFPI-2 was linked to poor prognosis and metastasis [18, 74, 84, 96]. Curiously, the opposite relationship was observed in clear cell carcinoma of the ovary (CCCO) where TFPI-2 was over-expressed and, together with CA125, represents a specific biomarker for preoperative prediction of CCCO [54]. Epigenetic alterations represent the main mechanisms implicated in the down-regulation of TFPI-2 expression although, to a lesser extent, 7q deletion involving the *TFPI-2* locus may also be responsible. Overall, there is scant data in the literature on the effect of *TFPI-2* gene mutations on cancer development. Deletion of the *TFPI-2* gene and as a result, a complete lack of TFPI-2 protein expression has been implicated in the genesis of uterine leiomyoma and prostate cancer [97–99].

Silencing of the *TFPI-2* gene is largely regulated by epigenetic mechanisms (without changes in the DNA sequence). Most often, TFPI-2 expression is down-regulated by hypermethylation of CpG islands located in the promoter region near the transcription start site. Silencing of the *TFPI-2* gene may also be regulated by chromatin remodeling via chemical modifications of histones which prevent transcription factors from binding to DNA regulatory sequences.

Additionally, down-regulation of *TFPI-2* is dependent on other mechanisms involving non-coding RNAs which control the structure of chromatin, such as small non-coding RNAs – microRNAs (miRNAs) or long non-coding RNAs (lncRNAs). miRNAs are involved in both activation and repression of transcription, as well as alternative splicing processes. miRNAs function primarily by binding to the 3’-UTR of their target mRNAs and exert post transcriptional gene silencing by degrading mRNA or inhibiting translation. On the other hand, lncRNAs are engaged in nucleosome positioning and chromatin loop formation, as well as being involved in *de novo* DNA methylation.

Over the past several years, nuclear poly-ADP-ribosylation (post-translational modification) has been recognized as an epigenetic marker, due to its influence on DNA methylation, histone modification, and chromatin remodeling; all the core histones may be modified by ADP-ribose, but primarily by mono-ADP-ribosylation or short oligomers of ADP-ribose [100].

In CRC, genome-wide methylation analysis has identified a core set of hypermethylated genes including *TFPI-2*. There exist a subset of CRCs, called CpG island methylator phenotype (CIMP), that result from the inactivation /silencing of several suppressor genes [101]. The highly-methylated *TFPI-2* gene has frequently been detected in advanced well-differentiated CRC [48]. Moreover, the *TFPI-2* methylation level has been found to be high and frequent in the peritumoral mucosa of cancer patients and aberrant methylation of *TFPI-2* has been identified in CRC development, during transition from its precursor-adenoma to cancer [94].

Ruixue Lei et al. identified two eligible methylation markers, *TFPI-2* and *SDC2* (syndecan 2) genes in CRC samples [102]. Interestingly, they observed a positive correlation between the *SDC2* and *TFPI2* methylation levels and microsatellite instability (MSI) scores in MSI-H (high microsatellite instability) CRCs. High-methylated *SDC2* or *TFPI2* were found in more than 95% of CRC patients [102].

Another study further proposed that methyl-CpG binding domain protein 3 (*MBD3*) which promotes HCC progression and metastasis through the negative regulation of *TFPI-2* may be a novel marker, facilitating early diagnosis, as well as constituting a promising therapeutic target in HCC. *MBD3* may suppress transcription of *TFPI-2* which results in HCC progression and metastasis. The process evolves through nucleosome remodeling and deacetylation of deacetylase (NuRD) complex, leading to reactivation of the matrix metalloproteinases (MMPs) and phosphoinositide 3-kinase/protein kinase B (PI3K/AKT) pathways. *MBD3*-knockdown Huh7 hepatoma cells exhibited suppressed invasive potential and proliferative rate. Interestingly, transfection of *MBD3*-knockdown cells resulted in significantly increased proliferative and invasive capability [103].

The loss of *TFPI-2 *expression, as a result of promoter hypermethylation, was found to be a key event for oral tumorigenesis and metastasis of OSCC [95, 104]. Silenced *TFPI-2* was re-expressed in OSCC cell lines after 5-Aza-dC treatment, suggesting its effectiveness in OSCC therapy. The demethylating compound, which restores tumor suppressor functions, also showed effects in prostate cancer [105] and HCC [50]. Wong et al. (2007) demonstrated that the *TFPI-2* gene in HCC is frequently silenced not only by promoter methylation, but also as a result of histone deacetylation, and that expression of the gene in HCC cell lines could be restored by a combined treatment with 5-Aza-dC and histone deacetylase inhibitor – tricostatin [50]. The proliferative and invasive potential of HCC cells can be robustly inhibited by ectopic *TFPI-2* over-expression. Further work on HCC has demonstrated that methylation of *TFPI-2* is linked to a high risk of advanced tumor stage, early tumor recurrence, poor prognosis, and that it could be a potential negative prognostic biomarker in patients with HCC after hepatectomy [106]. Similarly, in GBM, high-methylated *TFPI-2* contributed to the aggressive phenotype of brain tumor cells and might constitute a negative prognostic marker [18, 78]. George J. et al. (2007) reported that restoration of *TFPI-2* can inhibit tumor progression in a variety of ways, through regulating the activity of MMPs and plasmin or inducing both intrinsic and extrinsic caspase-mediated pathways leading to apoptosis in U-251 cells. The results may point to a new potential cancer gene therapy based on recombinant adeno-associated viral vector (rAAV) mediated TFPI-2 re-expression.

*In vitro*, TFPI-2 has been reported to suppress breast cancer (BC) cell proliferation and invasion [107]. Decreased expression of *TFPI-2* by aberrant methylation in the promoter region has been proposed as a mechanism contributing to cancer progression and recurrence, and to poor survival in BC patients. Thus, TFPI-2 mRNA levels in malignant breast tumors were found to be reduced compared to those in normal breast tissues [74]. Further evidence supporting a tumor suppressor role of *TFPI-2* was provided by Andresen MS et al. (2020) who reported a positive correlation between miR-494 and TFPI-2 mRNA levels in BC tumors. They found that miR-494 increased the TFPI-2 mRNA levels in BC cells line MCF-7, probably via indirect regulation of interactions between miR-494 and AHR as well as ELF-1 transcription factors, which can bind to the 5’-untranslated region (UTR) of *TFPI-2*. Consequently, *TFPI-2* has been proposed as a positive prognostic marker in breast cancer [108].

Li M. et al. (2019) suggested that mono-ADP-ribosylation of point mutation H3R117 colon adenocarcinoma LoVo cells up-regulated methylation of *TFPI-2* via TET1 (ten-eleven translocation family member). Since *TFPI-2* hypermethylation is an early event in tumorigenesis, the authors concluded that selectively targeting ribosylation of H3R117 deficiency may block tumorigenesis in CRC [109].

Gao F et al. (2017) showed that miR-130a was up-regulated in both hemangioma tissue and cell lines. miR-130a binds directly to the 3’-UTR of TFPI-2 mRNA. The authors demonstrated that the miR-130a inhibitor increased the expression of TFPI-2. The results indicate that suppression of miR-130a (functioning as an oncogene) significantly inhibited the growth of hemangioma by influencing TFPI-2 mRNA levels and blocking the focal adhesion kinase (FAK)/PI3K/Rac1/anti-mouse double minute (mdm2) [110].

TFPI-2 was found to be inversely correlated with miR-616 in a study performed by Ma et al. (2011). They observed that TFPI-2 is significantly down-regulated by microRNA-616 (miR-616) in androgen-independent prostate cancer cell lines compared with androgen-dependent prostate cancer cell lines or normal prostate cells and may contribute to disease progression and resistance to treatment in prostate cancer. Thus, it appears that miR616 induces androgen – independent growth of prostate cancer cells by suppressing expression of TFPI-2 [111].

In GC, epigenetic silencing of the *TFPI-2* gene is dependent on both promoter methylation and miR-27a-3p regulation and is linked to poor clinical outcome [96]. miR-27a-3p—specific inhibitor demonstrates a significant tumor suppressor function similar to that of *TFPI-2* itself. The anti-tumoral activities are completely abolished by *TFPI-2* knockdown.

Epigenetic silencing of *TFPI-2* has also been described in cervical cancer [112]. Not only was it found that hypermethylation of the *TFPI-2* gene occurred exclusively in tumor cells, but levels of TFPI-2 mRNA were also markedly reduced in tumor-associated fibroblasts, despite the *TFPI-2* gene being unmethylated. This suggests that apart from hypermethylation, an alternative mechanism must be responsible for its decreased mRNA expression, likely regulated by microRNAs. In particular, miR-23a, implicated in inhibiting TFPI-2 translation, was found to be highly correlated with the changes in TFPI-2 proteins. Transfection of miR-23a mimic substantially decreased TFPI-2 protein expression while miR-23a inhibitors gave rise to increased TFPI-2. Thus, down-regulation of miR-23a expression by HPV in cancer cells caused TFPI-2 expression to be silenced by promoter methylation. Equally, miR-23a activity in fibroblasts, unaffected by HPV, appears also to have inactivated the TFPI-2 protein expression.

Numerous studies have shown that lncRNAs play a crucial role in various types of cancer and their dysregulation is associated with the development, invasion and metastasis of tumors [113–116]. In 2017, Gao and colleagues identified a novel lncRNA, TFPI2AS1, which is an antisense transcript of *TFPI-2 *gene, and went on to explore the relationship between TFPI2AS1 and *TFPI-2*. Down-regulation of TFPI2AS1 by siRNA transfection promoted proliferation and migration of NSCLC cells by facilitating the G1/S transition, while over-expression of TFPI2AS1 had the opposite effect. LncRNA silencing resulted in decreased expression of the tumor suppressor TFPI-2, while overexpression of TFPI2AS1 increased TFPI-2 mRNA and protein levels as expected. On this basis it may be inferred that TFPI2AS1 expression is positively associated with TFPI-2 expression and the effects of lncRNA on cell proliferation and migration in cell lines may be achieved by means of boosting TFPI-2 expression. This observation may provide a novel direction for future research and treatment of NSCLC [110, 117].

The antisense lncRNA ArfGAP with GTPase domain, Ankyrin repeat and PH domain 2 Antisense 1 (AGAP2-AS1) exhibits oncogenic properties in several cancers, including GBM. It binds to methyltransferase, EZH2, and histone demethylase, LSD1, which in turn epigenetically silences TFPI-2. Over-expression of AGAP2-AS1 promoted GBM cell proliferation and invasion and positively correlated with poor prognosis in GBM patients. The tumor-suppressive effects mediated by AGAP2-AS1 knockdown were largely reversed following down-regulation of *TFPI-2*. Taken together, the roles and mechanisms of AGAP2-AS1 are likely to provide novel insights for GBM therapy [118].

#### TFPI-2 as a regulator of other pathways

TME plays a fundamental role in cancer development. Extracellular metalloproteinases are crucial regulators of TME functions. One of them, a disintegrin and metalloproteinase with thrombospondin type 1 motif 1 (ADAMTS1) has been reported to alter TFPI-2 binding properties as well as its location, thereby bringing about changes in its function [119]. It is well known that ADAMTS1 plays a supporting role in cancer development by eliciting structural changes in the TME [120].

N^6^-methyladenosine (m^6^A) RNA methylation, a common modification of mRNA, plays a role in the regulation of ECM in PC. ECM in this tumor type is profuse and m^6^A related targets are functionally concentrated in the ECM. m^6^A – related genes are regulated by FTO (fat mass and obesity – associated gene). Silencing of FTO results in up-regulation of TFPI-2 expression (i.e. through restoring m^6^A/YTHDF1 mediated stability of TFPI-2 mRNA), and inhibits PC proliferation, invasion *in vitro,* and tumor growth *in vivo* [121].

TWIST1 is a member of the transcription factor family that promotes epithelial-mesenchymal transition (EMT), cell migration and cancer invasion. It plays a role in breast cancer progression and has been suggested to be a prognostic marker. The effect is associated with up-regulation of integrin α5 expression. In BC, low levels of TFPI-2 expression correlate with tumor growth, metastasis and pathological stage. Functional assays have shown that TWIST1 reversed the inhibitory effects of over-expressed TFPI-2 on cancer. TFPI-2 suppresses BC growth by initiating blockade of TWIST1-mediated integrin α5 expression [74].

lncRNAs have been shown to interact with DNA, while other RNAs and proteins are known to regulate cellular processes. *MEG8* (at the 14q32 locus) is induced in ischemic heart disease and has been hypothesized to be an epigenetic regulator involved in angiogenesis after ischemia or endothelial function expression. Investigation with human umbilical vein ECs (HUVECs) revealed that silencing of *MEG8* resulted in reduction of total sprout length (angiogenic sprouting) and proliferation while migration was unaffected. Of interest, *MEG8* silencing resulted in a fivefold increase in TFPI-2 mRNA. Specifically, inhibitory histone modification H3K27me3 at the *TFPI-2* promoter was observed to be reduced as a result of *MEG8* silencing. Consequently, *TFPI-2* silencing partially reversed reduced angiogenic sprouting capacity without any effect on cell proliferation with silenced *MEG8*. Interfering with the MEG8/TFPI-2 axis might lead to an improvement in angiogenesis following ischemia. Therefore, interventions affecting MEG8/TFPI-2 might have potential as a therapeutic approach in the treatment of ischemic incidents [92]. In addition, Terashima et al. (2018) showed that *MEG8* plays a role in EMT in lung cancer and PC cells by regulation of gene expression. Mechanistically, *MEG8* suppressed microRNA-34a and microRNA-203 gene expression, and up-regulated SNAI1 and SNAI2 transcription factors, further limiting expression of cadherin1 (CDH1)/E-cadherin. Furthermore, *MEG8*, together with EZH2, increased histone 3 (H3) methylation in Panc1 (pancreatic cancer cells). EZH2 was shown to trimethylate H3K27 to induce transcriptional silencing. After silencing of *MEG8*, there was an 80% reduction in the inhibitory potential of H3K27 trimethylation [122]. EZH2 has been shown to silence the expression of *TFPI-2* in GBM [118]. Summing up, loss of *MEG-8* reduces H3K27me3-mediated decrease in *TFPI-2* expression [122].

Intriguingly, *TFPI-2* was shown to exert a pro-invasive effect on human HCC cells, according to Neaud et al. (2000). The study demonstrated that liver myofibroblasts promote *in vitro* invasion of HCC cells, not only through the hepatocyte growth factor/urokinase/plasmin—involved mechanisms, but also by synthesis of *TFPI-2*. Moreover, HCC cells transfected with TFPI-2 expression vector acquire invasive properties. HCC cells have the potential to express factor VII and tissue factor. It has been suggested that TFPI-2 induces invasion of HCC through binding to tissue factor-factor/VIIa complex, since the specific anti-fVII antibody abolished TFPI-2 induced invasion of HCC cells [66].

Recently, new research has shed more light on TFPI-2 function within the context of poly (ADP-ribose) polymerase 1 (PARP1) activity. PARP1 is involved in DNA damage repair and the pathogenesis of a range of diabetic complications, i.e., cardiomyopathy, retinopathy, neuropathy and vasculopathy. *PARP1* knockout in Type I diabetes mellitus (T1DM) mouse models underwent ligation of the carotid artery to induce neointimal hyperplasia. The deletion of *PARP1* was associated with inhibition of neointimal hyperplasia. Further experiments revealed that hyperglycemia-induced PARP1 promotes diabetic neointimal hyperplasia via down-regulating TFPI-2 (which normally suppresses proliferation of vascular smooth muscle cells). PARP1 probably works as a negative transcription factor which enhances *TFPI-2* promoter methylation. Interestingly, the researchers concluded that hyperglycemia silences *TFPI-2* via PARP1*-*mediated *TFPI-2* promoter methylation [123].

It has been hypothesized that the role of TFPI-2 depends on its cellular localization and is multi-directional. TFPI-2 is thought to interact with actinin-4 and myosin-9 in the cytoplasm, with AP-2α in the nucleus and with the ERK-signaling pathway, affecting nuclear localization of pERK1/2, which, as a result, causes inhibition of tumor cell motility, proliferation and invasion [107]. Studies on BC have revealed that over-expression of TFPI-2 limits phosphorylation of EGFR/ERK1/2 and decreases pERK1/2 translocation into the nucleus, which results in a reduction in cell proliferation. Further tests demonstrated the interaction of TFPI-2 with actinin-4 and myosin-9 [107], that did not change in accordance with TFPI-2 expression levels. Biochemical analysis showed that for interaction with actinin-4, full-length TFPI-2 is required, but for proper binding to myosin-9, either full length or only N-terminus + KD1 regions of TFPI-2 are sufficient. Further, TFPI-2 over-expression inhibits the migration and invasiveness of BC cells. Interaction of TFPI-2 with actinin-4 or myosin-9 in cytoplasm and regulation of ERK1/2 signaling may influence cell motility and invasion potential [107].

TFPI-2 exhibits both cytoplasmic and nuclear distribution, and interact with prosaposin (PSAP) blocking its preinvasive activity. Moreover, a co-immunoprecipitation and immunofluorescence study showed that PSAP interacts with the Kunitz-type domain 2 (KD2) region of TFPI-2, which could inhibit the invasion-promoting effects of PSAP in human HT1080 fibrosarcoma cells [16]. Furthermore, TFPI-2 can be internalized in various cells and transported to the nucleus by the importin system. In a study in HT-1080 cells, exogenous TFPI-2 was taken up and distributed to the cytoplasm and nucleus [124]. It appeared that nuclear localization of TFPI-2 required an NLS sequence located in its C-terminal tail, and TFPI-2 and importin-alpha complexes were co-immunoprecipitated from HT-1080 cells lysates demonstrating that the importin system is engaged in the transportation of TFPI-2 into the nucleus [124]. Furthermore, translocation of TFPI-2 into the nucleus allows it to interact with transcription factors, such as AP-2α and to modulate the expression of several genes, i.e., by causing silencing of the *MMP-2* gene [85].

Another mechanism facilitating EMT and promoting tumor growth and metastasis involves transmembrane serine proteases such as transmembrane protease serine 4 (*TMPRSS4*). Methylation of the *TFPI-2* promoter is correlated with increased expression of *TMPRSS4*, which subsequently promotes oncogenesis in NSCLC [125]. *TMPRSS4* has been suggested as a novel oncogene, as well as a prognostic biomarker, whose action may be dependent on pathways including NF-κB [126], the TMPRSS4/SLUG-TWIST1/SOX2 axis [127], MAPK and AKT [128], and ERK 1/2 [129] signaling.

The antitumor effects of retinoids acting via the retinoid acid receptor α (RARα) have been reported, along with the identification of factors engaged in the transcriptional regulation of TFPI-2 in HCC [130]. All-*trans*-retinoic acid (ATRA), used in the study, caused over-expression of TFPI-2 in HuH7 HCC cell-lines which was critical for ATRA-associated inhibition of HuH7 cell invasion. Activation of the *TFPI-2* promoter was increased by the musculo-aponeurotic fibrosarcoma oncogene homolog B (MAFB) via RARα and decreased by MAFF. MAFB exerts antagonistic effects towards MAFF in the context of inhibiting TFPI-2. ATRA suppressed HuH7 cell invasion which was inhibited by the knockdown of RARα or MAFB and the inhibitory effect was strengthened by MAFF. TFPI-2 expression was significantly down-regulated in HCC and this effect was possibly dependent on the low expression of RARβ and MAFB. In the low MAFB and high MAFF group of patients with HCC, the disease-free survival (DFS) was the shortest, which supports the role of the MAFB and MAFF in regulation of retinoid-related tumor suppressor genes such as *TFPI-2*. Since supplementation of retinoids may be helpful in patients with hepatitis C virus-related HCC, the vital part of treatment may be the activation of RARα and therefore up-regulation of TFPI-2 [130].

There have been attempts to modify the amount of TFPI-2 expressed in various cell types and cancer. In this context it is worth mentioning the study by Guan et al. (2022). In mice with streptozocin-induced diabetes, up-regulation of TFPI-2 was observed in renal cortex, together with impaired renal function. Ameliorated kidney functions and reduced renal fibrosis were observed following single-dose injections of AAV2 carrying TFPI-2 silencing shRNA. Similarly, hyperglycemia-induced endothelial-mesenchymal transition was limited in the absence of TFPI-2. The study demonstrated that TFPI-2 modulated TGF-β2/Smad signaling via inhibiting its negative regulators SMURF2 (SMAD ubiquitination regulatory factor-2) and SMAD7. TFPI-2 decreases SMAD7/SMURF2 complex formation, abolishes recruitment of SMURF2 to TGFBR1/2 and further ubiquitination and degradation of TGFBR1/2. Consequently, TFPI-2 promotes TGF-β/SMAD2/3 signaling. The deficiency of TFPI-2 was associated with inhibition of high glucose-dependent cell apoptosis and also TGF-β2-induced endothelial-mesenchymal transition. The opposite effects were observed when TFPI-2 was over-expressed [131]. The precise effects of TFPI-2 on TGF-β/SMAD2/3 signaling and on diabetic nephropathy require further *in vivo* research. Moreover, TGF-β2 also functions through other signal transduction pathways such as MAPK.

**Fig. 3:**
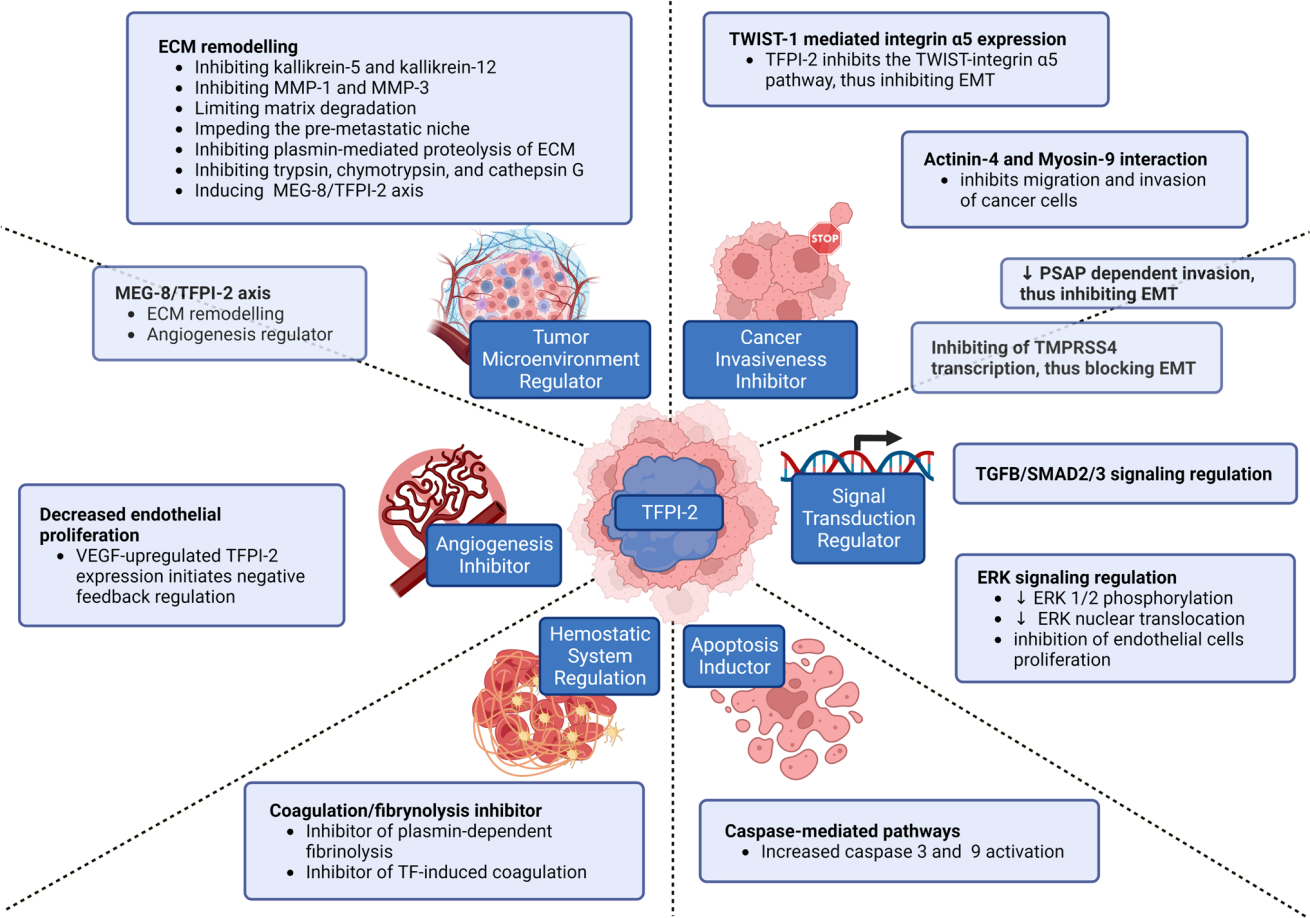
Mechanisms of TFPI-2 interaction with cancer. Created with BioRender.com. (EMT—epithelial-mesenchymal transition; ERK—extracellular signal-regulated kinase; MEG8—Maternally Expressed Gene 8; MMP-1—matrix metalloproteinases 1; MMP-9—matrix metalloproteinases 9; PSAP—prosaposin; SMAD—Mothers against decapentaplegic; TF—tissue factor; TFPI-2—Tissue factor pathway inhibitor-2; TGFB—transforming growth factor beta; TMPRSS4—transmembrane protease serine 4; TWIST1—Twist-related protein 1; VEGF—vascular endothelial growth factor)

TFPI-2 interactions with cancer are summarized in Fig. 3.

## Future perspectives: TFPI-2 as a potential biomarker

There are several studies reporting the significance of TFPI-2 evaluation in the prognosis of cancer. An aberrantly spliced variant of *TFPI-2* mRNA up-regulated in cancer has been identified [31]. Various studies have emphasized the methylation status of the *TFPI-2* gene promoter. For example, in non-metastatic NSCLC patients, methylation-specific PCR (MSP) showed methylation of *TFPI-2* in 27.1% of patients, of whom 47.2% had stage III tumors. Analyses suggested that methylation of *TFPI-2* is an independent negative prognostic factor in NSCLC patients and is associated with poor overall survival, based on multivariate analysis (p = 0.013) [45]. Moreover, in the case of *TFPI-2* silenced NSCLC patients, about half were lymph node*-*positive [44].

Interestingly, aberrant methylation of *TFPI-2* gene promoter may be assessed in stools and may serve as a novel additional marker for the early detection and prognosis of CRC [132]. The newest research has brought additional knowledge demonstrating that *TFPI-2* and *SDC2* (another methylation-based marker in CRC) may form three methylation phenotypes of CRC: High-methylation/High-methylation (HH), High-methylation/Low-methylation (HL), and Low-methylation/Low-methylation (LL) with regard to *SDC2* and *TFPI-2* methylation status, respectively. In the HL group, CRCs often originated from the left side, whereas in the HH group, there is more microsatellite instability and mutation load and almost all *BRAF* mutations were noted in the HH group with older patients belonging to the HH group [102]. The study found that a combination of both *TFPI-2* and *SDC2* gene targets appeared to be most effective in detecting CRC, which provides the opportunity for introducing noninvasive stool- or blood-based minimally invasive detection techniques in the future [102].

The diagnostic value of *TFPI-2* methylation status analysis has been demonstrated in several studies. *TFPI-2* as a stool- or blood-based DNA noninvasive biomarker has been shown to have high sensitivity and specificity in CRC screening [132–138].

Likewise, correlation analysis between TFPI-2 expression and breast cancer clinicopathologic features has shown a significant relationship between tumor size, histologic grade, lymph node status, and blood vessel invasion in BC. Patients with higher levels of TFPI-2 had longer DFS. Patients with negative to low TFPI-2 expression showed more progression and recurrence after BC surgery [139]. Further, in HCC after hepatectomy, the methylation status of *TFPI-2* is of prognostic value. *TFPI-2* methylation, detected in 44.9% of HCC samples, was correlated with higher TNM and both shorter DFS and OS (*p* = 0.00 and *p* = 0.002, respectively), and importantly, it was found to predict early tumor recurrence after hepatectomy [106].

Current research has brought to light some interesting results in this area: the higher the percentage of methylation of *TFPI-2* and *NDRG-4* (N-Myc downstream-regulated gene-4) promoters, the higher the clinical stage of CRC. The methylation rate for the two genes was 20% and 22.8% for stage I CRC, which increased steadily reaching 75% and 63%, respectively, for stage IV CRC. In contrast, the percentage of methylation was only 11.56% and 12.23% in control groups, respectively, for the two genes promoters. Methylation tests in peripheral blood mononuclear cells may be a sufficient non-invasive screening method of high sensitivity and specificity [138]. Furthermore, low expression of TFPI-2 has been related to poor prognosis in GC [96].

Likewise, a genome-wide DNA methylation analysis of malignant melanoma samples highlighted a subtype of melanoma with higher methylation rates. Significant hypermethylation involved 27 genes, among which *TFPI-2* was found to be the most frequently hypermethylated. Epigenetic aberration was correlated with significantly higher thickness of tumor and more progressive phenotype. *In vitro* studies on melanoma cell lines, CHL-1 and G361, confirmed the above results, showing that TFPI-2 knockdown was associated with a marked increase in cell proliferation and invasion [140].

TFPI-2 has been proposed as a serodiagnostic marker of epithelial ovarian cancer (EOC), in particular, CCC. While TFPI-2 is down-regulated in most cancer types, it is over-expressed in CCC. It has been hypothesized that CCC cells promote TFPI-2 expression and secretion in order to survive in unfavorable conditions. TFPI-2 secreted by CCC may be detected in serum and represent the level of over-expression of TFPI-2 in CCC [14]. The latest data indicate that TFPI-2 is an accurate ovarian cancer marker, with findings similar to Risk of Ovarian Malignancy Algorithm (ROMA) for discriminating between malignant and benign ovarian tumors. The best results were observed for a combination of TFPI-2 and ROMA [141].

The lncRNA AGAP2-AS1 may also bear a prognostic value for certain types of tumors [142]. It has been postulated that AGAP2-AS1 has the potential to be a biomarker in NSCLC [143] and anaplastic glioma [144]. The level of AGAP2-AS1 in NSCLC was strongly correlated with the stage of cancer and lymph node metastases. Patients with higher expression of AGAP2-AS1 had shorter OS than patients with lower expression of AGAP2-AS1 [143].

The expression of AGAP2-AS1, as well as three other lncRNAs (TPT1-AS1, LINC01198 and MIR155HG) were studied in different grades of anaplastic gliomas (144). An analysis of these lncRNAs, allowed the authors to divide patients into low-risk and high-risk prognostic groups. The low-risk group was characterized by a longer OS (corresponding to patients with Grade 2), while higher-risk patients had a much shorter OS (Grade IV). The authors concluded that a signature based on the expression of the four lncRNAs has prognostic value for anaplastic glioma and may have clinical implications.

## Future perspectives: TFPI-2 as a potential target of anticancer therapy

Numerous studies have addressed the possibility of using TFPI, and in particular, TFPI-2, as a target in gene therapy for cancer, including adenoviral mediated or nanoliposome-associated delivery of TFPI-2 or inducing upregulation of TFPI-2 expression.

There have been *in vivo* ‘trials’ in nude mice bearing laryngeal squamous cell carcinoma using adenoviral-mediated (Ad)-*TFPI-2* gene transfer. The gene therapy involved injecting adenoviruses carrying TFPI-2 directly into the tumor, which led to reduced tumor volume and weight (*p* < 0.01) and increased apoptosis [27]. Similarly, in gallbladder carcinoma models, adenovirus-mediated gene transfer of *TFPI-2* (Ad5-*TFPI-2*) was used to restore the expression of *TFPI-2* in cell lines of gallbladder carcinoma and in xenograft tumors, which again led to increased apoptosis both *in vitro* and *in vivo* [145].

Similarly, *in vitro* experiments with over-expression of *TFPI-2* in KYSE450, KYSE510, YES2, and EC9706 cells has demonstrated significantly lower invasiveness. Furthermore, in esophageal cancer xenografts (EC9706), tumor growth and invasion as well as lung metastasis were all inhibited with *TFPI-2* over-expression. Recombinant TFPI-2 protein also limited the activity of MMPs and angiogenesis and further significantly inhibited xenograft growth and metastasis [146].

TFPI-2 protein can also be delivered to tumors via various nanocarriers. For example, folic acid-targeted pH and redox dual stimulation-responsive β-cyclodextrin—hyperbranched poly(amido amine)s (FA-DS-PAAs) nanocarriers were used to deliver docetaxel and TFPI-2 in nasopharyngeal carcinoma (NPC) *in vitro* and *in vivo*. The results showed that the co-delivered TFPI-2 enhanced docetaxel toxicity and caused more pronounced apoptosis and inhibited cell invasion in comparison to monochemotherapy. Notably, the hepatotoxicity of the nanomedicine-mediated co-delivery of TFPI-2 and docetaxel was lower than that observed for docetaxel [147].

TFPI-2 was also co-delivered in folic acid (FA)-targeted magnetic nanocomposite together with cisplatin or CDDP (FA-MNP/CDDP/TFPI-2) [148]. Studies in nasopharyngeal carcinoma HNE-1 cells *in vitro* and *in vivo* (BALB/c nude mice xenografted tumor model) showed that co-delivered drugs induced more pronounced apoptosis than single treatment with either CDDP or TFPI-2. FA-MNP/CDDP/TFPI-2 exhibited good gene delivery efficiency and revealed a significant response in FA-positive cancer cells. FA-MNP/CDDP/TFPI-2 also efficiently inhibited nasopharyngeal cancer growth *in vivo*. Additionally, the authors suggested the use of the nanocomposite in magnetic hyperthermia and magnetic targeted therapy with the aid of the MNP carrier [148].

Recently, a novel oxidized, sulfated, non-anticoagulant ultra-LMWH (S-NACH) has emerged. S-NACH is a sulfated form of LMWH without anti-factor Xa activity, which does not demonstrate anti-IIa activity, thus having limited or no effect on systemic coagulation. This novel form of LMWH has enhanced ability to bind to ECs and promote release and activity of TFPI [149]. *In vitro* studies with human acute leukemia cells (K562) and human PC cells (SUIT2) demonstrated that S-NACH causes up to a threefold increase in TFPI-2 levels (released from human ECs) within 3 h compared to enoxaparin. Also, S-NACH, at a dose of 20 mg/kg subcutaneously, did not change bleeding time in comparison with tinzaparin and enoxaparin at 5 mg/kg. These findings suggest that S-NACH may be a promising tool in enhancing TFPI-1 and -2 activity, in cancer prevention and also non-cancer associated thrombosis treatment with much reduced risk of bleeding complications [149].

Recently, another regulatory mechanism engaging TFPI-2 has been described. In small cell lung cancer (SCLC), expression of lysine specific demethylase 2 (LSD2) and DNA methyltransferase 3B (DNMT3B) is increased while TFPI-2 expression is decreased. Simultaneously increasing the expression of LSD2 and TFPI-2 in small cell lung cancer was found to reduce its proliferation. Mechanistic studies suggested that LSD2 indirectly suppressed TFPI-2 expression via DNMT3B or via demethylation of H3K4me1 in the promoter region of *TFPI-2* [150].

The E2F5/p38/SMAD3 signaling axis has been found to be deregulated in prostate cancer. E2F5, a dual-function transcription factor was shown to be a potential risk factor in prostate cancer. E2F5 appears to trigger *TFPI-2* silencing and promotes MMP2 and MMP9 expression. Studies have shown that TFPI-2 is a downstream target for E2F5. In biopsies from individuals with prostate cancer, E2F5 as well as MMP-2 and MMP-9 levels were found to be elevated in comparison with benign prostate specimens. Artemisini, a natural anti-malarial compound inducing G1 cell-cycle arrest, repressed E2F5 together with MMP-2 and MMP-9 while upregulated TFPI-2, leading to inhibition of prostate cancer cell aggressiveness [151].

## Conclusions

For decades, components of the coagulation system have been studied in the context of cancer development. In particular, TFPI-2, a serine protease inhibitor with multi-faceted effects on tumor growth and metastasis, has attracted interest. There is a growing body of evidence to suggest that TFPI-2 functions as a tumor suppressor, plays a role in regulatory mechanisms, and affects coagulation, angiogenesis, ECM degradation and expression of many other genes via epigenetic mechanisms. TFPI-2 promotes cell apoptosis and takes part in intracellular signal transduction. It is noteworthy that TFPI-2 acts not only in the extracellular environment, but also in the cytoplasm and in the nucleus. TFPI-2 expression is reduced in cancer tissue, and in numerous cancer types its low-expression correlates with the advanced stage or grade of the tumor. Thus, TFPI-2 may be a promising theranostic biomarker for cancer. Nonetheless, the role of TFPI-2, which expression appears to be elevated in several other cancers such as CCC of the ovary and HCC, is incompletely understood. Therapeutic attempts to restore or upregulate TFPI-2 expression in cancer are still in the early stages and further systematic studies are required.

## Data Availability

No datasets were generated or analysed during the current study.

## Abbreviations

AAV: Adeno-associated virus
ADAMTS1: ADAM Metallopeptidase with Thrombospondin Type 1 Motif 1
AGAP2-AS1: ArfGAP with GTPase domain, Ankyrin repeat and PH domain 2 Antisense 1
AKT: Protein kinase B
ATRA: All-trans-retinoic acid
CCC: Clear cell carcinoma
CDH1: Cadherin1
CIMP: CpG island methylator phenotype
CM: Cutaneous melanoma
COX-2: Cyclooxygenase-2
CRC: Colorectal cancer
DFS: Disease-free survival
DNMT3B: DNA methyltransferase 3B
ECM: Extracellular matrix
ECs: Endothelial cells
EMT: Epithelial-mesenchymal transition
EOC: Epithelial ovarian cancer
ERK: Extracellular signal-regulated kinase
EZH2: Zeste homolog 2
FA-DS-PAAs: Folic acid-targeted pH and redox dual stimulation-responsive β-cyclodextrin—hyperbranched poly(amido amine)s
FAK: Focal adhesion kinase
FA-MNP/CDDP/TFPI-2: Folic acid (FA)-targeted magnetic nanocomposite co-delivering cisplatin (CDDP) and TFPI-2
FGF-2: Fibroblast growth factor
FTO: Fat mass and obesity-related protein
fVIIa: Factor VIIa
fXa: Factor Xa
GBM: Glioblastoma multiforme
GC: Gastric cancer
HCC: Hepatocellular Carcinoma
HPV: Human papilloma virus
HUVECs: Human umbilical vein endothelial cells
IL-1β: Interleukin-1β
KD2: Kunitz-type domain 2
Ki67: Proliferative index
lncRNAs: Long non-coding RNAs
LSD1: Lysine specific demethylase 1
LSD2: Lysine specific demethylase 2
MAFB: Musculo-aponeurotic fibrosarcoma oncogene homolog B
MAFF: Musculo-aponeurotic fibrosarcoma oncogene protein F
MAGEA3: Melanoma-associated antigen 3
MAPK: Mitogen-activated protein kinase
MBD3: Methyl-CpG binding domain protein 3
MEG8: Maternally Expressed Gene 8
miRNAs: MicroRNA
MMPs: Matrix metalloproteinases
MSI: Microsatellite instability
NDRG-4: N-Myc downstream-regulated gene 4
NO: Nitric oxide
NPC: Nasopharyngeal carcinoma
NSCLC: Non-small cell lung cancer
OSCC: Oral squamous cell carcinoma
PAR-1: Protease-activated receptor-1
PARP1: Poly (ADP-ribose) polymerase 1
PI3K: Phosphoinositide 3-kinase
pKLK: Plasma kallikrein
PSAP: Prosaposin
rAAV: Recombinant adeno-associated viral vector
RARα: Retinoid acid receptor α
ROMA: Risk of Ovarian Malignancy Algorithm
SDC2: Syndecan 2
SMAD: Mothers against decapentaplegic
SMURF2: SMAD specific E3 ubiquitin protein ligase 2
S-NACH: Novel oxidized sulfated ultra-LMWH
T1DM: Type I diabetes mellitus
TET1: Ten-eleven translocation
TF: Tissue factor
TFPI-2: Tissue factor pathway inhibitor-2
TGFBR1/2: Transforming growth factor beta receptor 1/2
TME: Tumor microenvironment
TMPRSS4: Transmembrane protease serine 4
TNF-α: Tumor necrosis factor
TWIST1: Twist-related protein 1
UM: Uveal melanoma
VEGF: Vascular endothelial growth factor
VEGFR2: Vascular endothelial growth factor receptor-2
VM: Vasculogenic mimicry
